# Working with Mild Traumatic Brain Injury: Voices from the Field

**DOI:** 10.1155/2012/625621

**Published:** 2012-02-07

**Authors:** Page Walker Buck, Rebecca G. Laster, Jocelyn Spencer Sagrati, Rachel Shapiro Kirzner

**Affiliations:** ^1^Graduate Social Work Department, West Chester University, West Chester, PA 19383, USA; ^2^Graduate School of Social Work and Social Research, Bryn Mawr College, Bryn Mawr, PA 19010, USA; ^3^Direct Clinical Services, Survivors of Abuse in Recovery, Inc., Wilmington, DE 19803, USA; ^4^Specialized Health Services, Public Health Management Corporation, Philadelphia, PA 19102, USA

## Abstract

Mild traumatic brain injury (mTBI), also known as concussion, is an emerging public health issue in the United States. The estimated annual 1.2 million individuals who sustain this injury face a range of cognitive, psychological, and physical consequences for which rehabilitation protocols are being developed and implemented. On the frontlines of this developing area of rehabilitation work are professionals in a range of therapeutic settings whose practice wisdom has yet to be shared in the professional literature. This qualitative study aimed to fill this gap by exploring the experiences and insights of rehabilitation professionals serving mTBI patients in outpatient, civilian settings. An analysis of the qualitative data revealed five themes common in mTBI work, providing an in-depth look at this often challenging field of rehabilitation.

## 1. Introduction

In recent years, mild traumatic brain injury (mTBI) has emerged as a leading public health concern. Eighty-five percent of the 1.5 million traumatic brain injuries sustained by Americans every year are considered “mild” [1]. Mild traumatic brain injury, commonly known as concussion, is a serious neurologic condition that can have long-term cognitive, physical, emotional, and social consequences. Once believed to be a virtual rite of passage in childhood and sports, concussions are now largely understood by medical professionals to be a traumatic form of brain injury that requires careful diagnosis, management, and followup [2].

A traumatic brain injury (TBI) occurs when the brain experiences neurological or neuropsychological impairment as a result of an external trauma. This typically occurs when the head comes into direct contact with an object in situations commonly understood as “hitting your head” or “being hit in the head,” such as car accidents, contact sports, or domestic violence assaults. A TBI can also occur during the rapid acceleration or deceleration associated with whiplash in which movement of the brain against the inside of the skull causes trauma [3]. Perhaps the least understood mechanism by which a mild TBI can occur is a blast injury, typical in modern military combat and other conflicts. Even without blunt force or acceleration/deceleration, trauma to the brain can arise from the sheer energy of the supersonic waves created from blasts. Blast injuries are so prevalent among service members and veterans that mTBI has been named the “signature injury” of the conflicts in Iraq and Afghanistan [4].

A diagnosis of traumatic brain injury requires a classification of severe, moderate, or mild based on an evaluation of consciousness, amnesia and a Glasgow Coma score. Diagnostic technology, including CT scans and MRIs, can be effective in the diagnosis of severe and moderate injuries. These technologies, however, are not able to reliably detect mild injuries, making diagnosis of mTBI far more difficult [5]. In large part, diagnoses of mTBI are based on patient self-report. Kennedy and colleagues [6] found that although emergency room and outpatient medical professionals understood mTBI and its complications, they typically failed to assess for sequelae or make appropriate referrals. There have been significant advances, however, in diagnosis with the use of baseline testing. Many high school and college sports programs are requiring certain student athletes to get baseline neurological testing as a way to help diagnose and assess concussion [7]. The symptoms of mTBI can be cognitive, psychological, and physical. Cognitive symptoms include decreased processing speed, confusion or impaired cognition, problems with attention, impaired judgment, and amnesia, or other problems with memory, especially short-term memory. Psychological symptoms include irritability, anxiety, depression, and a change in personality. Physical symptoms include headaches, dizziness, diplopia, sensitivity to light or noise, tinnitus, and insomnia. These impairments often undermine academic and work performance, impacting employment, education, and relationships [8]. Generally symptoms resolve within a few weeks or months, yet for some, symptoms persist for long periods of time and can result in permanent disability [9].

Despite increases in awareness and concomitant increases in diagnosis, the development of evidence-based treatment and rehabilitation protocols for mTBI has lagged behind. A review of current practices [10] suggests that mTBI is largely treated using models developed for moderate and severe injuries, “despite strong evidence that they are distinct clinically and epidemiologically” and despite the fact that “rehabilitation designed for moderate and severe TBI has not been effective for mild TBI” [10, pp. 1590-1]. In 2007, the US Office of the Surgeon General and the Army Medical Department took an important step in addressing these treatment deficits with the establishment of a task force dedicated to developing best practices specific to treating mTBI in injured service members and veterans. Recommendations for best practices in both occupational and physical therapy have since been published for this population [11, 12].

Rehabilitation professionals who have direct experience with individuals diagnosed with mTBI have a wealth of knowledge of this injury from the hours they have spent with their patients. Despite having the most robust understanding of the current state of mTBI rehabilitation work, the experiences of mTBI professionals have yet to be included in published research. This qualitative study aims to fill this gap by giving voice to the practice experience of rehabilitation professionals working in mTBI.

## 2. Methods

This study was designed to gather information from rehabilitation professionals who had direct experience working specifically with patients diagnosed with mTBI. A participant recruitment letter, informed consent document, and interview protocol were developed and approved by West Chester University's Institutional Review Board prior to beginning data collection. Participants for the study were selected using nonrandom, snowball sampling techniques based on contacts with brain injury professionals. Participants were included in the study if they reported that they spent at least one quarter of their time with individuals diagnosed with mTBI.

In the spring of 2010, we conducted in-depth, semistructured interviews; either in person or via telephone, which lasted between 45 and 90 minutes each. Following a systematic process of informed and voluntary consent, we asked participants to describe their work with mTBI patients, beginning with the inquiry: “I'd like to hear about your experiences working with people with mild traumatic brain injuries.” To facilitate the data analysis process, all interviews were digitally recorded using either a handheld digital recorder or a secure, web-based conferencing program. Recordings were transcribed by a professional organization using encryption software.

Fifteen licensed professionals employed in civilian (nonmilitary), outpatient brain injury rehabilitation positions, including physical, vestibular, cognitive, occupational, speech, and neuropsychology, were interviewed. Participants included 4 men and 11 women who worked in rehabilitation settings in the Mid-Atlantic region of the United States, with an average of 14 years in the field, ranging from 2 to 23 years of experience. Many worked in settings that require them to see a range of patients, although none spent less than one quarter of their time with individuals diagnosed with mTBI.

The qualitative data obtained in the interviews were analyzed using a systematic process of analysis, coding, and discussion. This study was based in grounded theory techniques [13] as a way to explore the lived experiences of mTBI rehabilitation professionals. Distinct from theoretical approaches such as ethnography or phenomenology, a grounded theory approach seeks “to build theory rather than test it” [13, p. 13]. As such, our coding scheme had not been developed prior to conducting the interviews. We were not seeking to test or confirm certain theories about mTBI work, but rather to develop them. Although we had impressions about the data given our experiences in the interviews, we resisted labeling and categorizing until interviews were transcribed and ready for analysis. We developed an initial coding scheme through an inductive process of analysis using three interviews. Subsequent interviews were coded using the scheme by at least two people through a process of constant comparison. When differences arose in the coding, we engaged in discussion until a consensus could be reached. The final analysis resulted in five major themes, pulled from eight that initially emerged.

The design of this study creates specific limitations. A snowball sampling technique was used to identify individuals specifically working with mTBI patients. This necessarily means the sample is not representative of rehabilitation professionals in general. Further, the research participants in this study worked with patients who had been referred for rehabilitation services; thus, their experience is limited to a group of patients whose characteristics and experiences likely differ from those individuals who are also injured but who do not receive rehabilitation services. While we might hypothesize that individuals with mTBI who are not receiving rehabilitation services are generally less symptomatic, it is possible that the severity of their impairments has interfered with the process of obtaining treatment.

## 3. Results and Discussion

Rehabilitation specialists reflected on their experiences working with the mTBI population from various perspectives. They spoke about their own first-hand experiences with this type of rehabilitation work, giving examples of the course of treatment with specific patients. Often rehabilitation professionals included their patients' words as a way of conveying the more subtle, complex elements of the work. Sometimes they quoted specific patients, but more frequently they quoted what seemed to be an “aggregate voice” of many patients. This plurivocal, narrative style [14] added to the richness of our findings, allowing for multiple voices to come through.

Five primary themes emerged from our analysis of the qualitative interview data: prevalence of misdiagnosis and misinformation, unpredictability of prognosis, complexity of symptoms, impaired self-awareness, and invisible nature of injury. The themes are described below in the order that they typically emerged in the interviews and often how they emerged in the practitioner-patient relationship.

### 3.1. Prevalence of Misdiagnosis and Misinformation

One prominent theme to emerge from this study was the prevalence of misdiagnosis and misinformation that occurs with mTBI. Research participants reported that their patients often had earlier experience with misdiagnosis prior to their rehabilitation. Largely, this was a result of the fact that negative results on medical imaging such as CT scans and MRIs are typically interpreted as sufficient reason to rule out a TBI of any kind despite the fact that diagnostic technology is not yet sensitive enough to pick up these mild injuries [5].

*With mild traumatic brain injury, we really do not have a medical test that's accurate enough or detailed enough to really tell you which axons and dendrites might be sheared or which cell nuclei might be affected by the impact.*

*My patients say, “But, my MRI looks normal. They keep telling me my MRI is normal.”*

*They hear, “Based on testing, you do not have a bleed. The CT scan and the MRI is negative and so you should be able to return to work or school but just take it easy.”*

The lack of clear diagnostic tests that corroborate patients' symptomology contributes to feelings of frustration and misunderstanding. Rehabilitation professionals reported consistently hearing mTBI patients ask “Why am I having so much trouble if all my tests are normal?” In the face of symptoms that interfere with daily functioning, negative test results create a remarkably incongruous scenario, not only for patients but also for rehabilitation professionals.

Participants reported that their patients were also misinformed sometimes even by primary care physicians who made reassurances that mild brain injury is an uncomplicated event that resolves easily and quickly.

*Family practitioners typically will tell them, “Oh, it's four to six weeks. You'll be better. It'll be fine. It's all good. Just go home, rest, do not push yourself.”*

*They go to a doctor, and they do not get as much support. “You'll be fine. You look great. Your CAT scans are normal. You know, give it a month. It'll be fine.”*

Although important, sleep and rest do not necessarily produce the expected results in the type of atypical fatigue common in mTBI [15]. When efforts to “rest and take it easy” fail to produce expected recovery, even those who pursued further medical help were at times rebuffed.

*He was sent to a psychologist who was basically saying, “You need to just push yourself through the situation. You cannot give in to the anxiety. If you push yourself through and get to the other side, you'll see that you can survive it.”*

*So many of our patients have been transferred from one physician to another and they haven't really gotten a solid answer.*

Research participants were acutely aware that mTBI patients who had been referred for rehabilitation services generally need more than just rest or a motivational shove. Too often, they found that their patients were left to manage without proactive medical care to provide accurate information about the complexity or anticipated duration of symptoms related to mTBI.

### 3.2. Unpredictability of Prognosis

Participants reported being routinely asked to give their patients a prognosis. Once patients find mTBI professionals who understand their injuries and are sympathetic to their often-complicated experiences of seeking support, they are eager to get a reliable and predictable prognosis. In part, patient demand for a clear prognosis stems from the sociocultural expectation that doctors not only diagnose but also predict outcomes. Increasingly, medical professionals are under pressure to cure ailments in a society that resists acceptance of death, pain, and suffering [16]. Participants expressed frustration with the inability to satisfy their patients' understandable desire for a clear and simple prognosis.

*The hardest thing—a lot of people want an end date. They want an answer. They want—“When am I going to be back? When am I going to be healed? When am I going to be better?”*

*“You'll be better in three months.” You know, you cannot give that promise.*

*I think the most challenging thing is that nobody really has a crystal ball, your ability to heal is individual and it's based on so many factors.*

Although patients want to know when they will feel better or when their situation will significantly improve, the ability to predict the duration and even severity of symptomology with a diagnosis of mTBI can be difficult [9].

*You can have someone with a concussion rebound very quickly and get back to school or work without too much difficulty or you can have lingering symptoms that are very debilitating but they still have the same diagnosis.*

Understandably, many mTBI patients expect the prognosis to be consistent with their conceptualization of a mild diagnosis. As rehabilitation professionals know well, however, a diagnosis of mTBI does not always provide a clear treatment strategy in which quick and significant improvement is definite. Rehabilitation professionals reported needing to manage patients' disappointments given their expectations of recovery from a “mild” injury.

*We have to help people manage expectations. We want to be encouraging at the same time not be over-encouraging, so you do not want them to look through rose-colored glasses but you do not want to dash their hopes, either.*

*Across the board, patients do not realize that the side effects can be something that will be long lasting, and even permanent.*

The diagnostic use of the term mild can bear little relationship to the duration of symptoms or the time it takes for patients to actually feel symptom-free. The challenge for rehabilitation professionals is to keep patients motivated in the face of a complex and unpredictable injury.

### 3.3. Complexity of Symptoms

The complexity of symptoms associated with mTBI is another prominent theme that emerged from the analysis of the interviews with rehabilitation professionals. Respondents reported that this complexity adds to the challenge of working with mTBI survivors.

*It's challenging, and it takes a lot of time to make sure that you're managing all of their symptoms, because they have so many, and trying to make sure that you're addressing everything.*

*Even though the primary diagnosis is maybe a C5 spinal cord injury, the real problem is that they cannot remember anything and their attention is terrible, and they have all these behavioral problems.*

*You can see personality changes, memory changes, social changes, different cognitive things—like initiation, planning, organization.*

*All these realms are affected—sleeping, and sometimes vision, and they're dizzy, and they do not have balance.*

Rehabilitation professionals are not able to tell patients exactly what symptoms to expect because no two cases of mTBI present exactly the same. There are many different functional areas that can be affected by mTBI, including cognition, balance, vision, and emotional regulation [17].

The rehabilitation process can be further complicated by functional impairment, such as lack of attention, organizational deficits, or behavior changes. These types of symptoms can make it difficult for patients to be fully engaged in their own treatment. As one participant explained, “*Behavior becomes an issue, which interferes with their therapy*.” Persistent dizziness, double-vision, or migraines due to vestibular and neurological symptoms can also make it difficult for patients to complete, or even participate in, the therapeutic activities of any given appointment [12]. Explains one rehabilitation therapist, “*The exercises we're making them do make them dizzy.” *The nature of symptoms and the subsequent treatment complications are mutually exacerbating which can contribute to the complexity of rehabilitation with this population.

### 3.4. Impaired Self-Awareness

It is not uncommon for survivors of mTBI to be unaware of their own symptoms or the extent of their own deficits [18, 19]. Participants in this study reported that their patients often do not recognize the exact nature of their condition.

*I do not think some of them realize that they have changed—either the personality or their anger or their memory. They do not realize that it is all normal—it is all tied together.*

*Maybe some of them thought, “Maybe my balance is not that bad,” but then when we go through all the testing, it is bad …. And then as we talk with them, we might find more cognitive problems that they're having.*

*I think some of them do not understand the limits that they have. They look fine so they try to push, push, push.*

*They know they're not feeling good, but it's almost like until you start doing the evaluation and start talking to them about their symptoms, they do not fully really understand what they're experiencing.*

Participants also reported that some of their patients do not recognize their limitations or inabilities as symptoms. Other patients know of their symptoms but do not recognize the pervasiveness of the functional impairment.

*Some say, “I do not understand. I was able to do all of this before. I have all these other abilities. But it's still so hard for me to initiate getting out of bed in the morning or putting a plan together for my day.”*

*They did not understand that they'd be facing so many challenges across a multitude of fronts.*

Because the preexisting misunderstandings that patients have about concussion are often coupled with impaired appreciation of deficits, working with mTBI patients can be both challenging and frustrating.

 This lack of self-awareness is likely also a function of denial and minimization responses that are common and even vital in traumatic situations. Participants reported patients' resistance to acknowledging their deficits.

*They ask, “Why is this so hard? That should not be hard. I should be able to do that.”*

*People kind of get back to the workplace and they do not want to come out and say, “I cannot handle all this right now. I'm not the same person as I was.”*

*They realize how bad they feel when they try to get back to their regular routine; when they try to go back to work.*

Though initially useful as a temporary coping mechanism in the face of a traumatic event, downplaying functional limitations that result from mTBI can make it difficult for practitioners to fully address their patients' rehabilitation needs.

### 3.5. Invisible Nature of Injury

A lack of reliable diagnostic technology, coupled with the invisible nature of mTBI, places patients at risk of experiencing suspicion of their condition, much like any “unseen” injury or disease. The TBI professionals in this study reported that their patients often experience suspicion from family, friends, employers, and even medical professionals about the extent of their injury.

*I think because mild traumatic brain injury individuals look completely normal, even their family and their friends say. “What's wrong with them? Why cannot they do the things they did before?”*

*People will be told by their doctors, their spouses, their family, “It's all in your head.”*

*They do not want to say anything because they might think that they're kind of making it up.*

The invisible nature of this injury is specifically difficult in employment settings. Participants shared multiple stories of patients who had experiences with employers and coworkers who were suspicious of their injuries.

*One patient told me that people thought he was just trying to make a buck by being on a worker's comp.*

*A lot of bosses, they brush it off, like, “You're walking, you're talking, you're good.”*

*They think that the person is being lazy, that the person's not motivated, that they purposefully are not being productive.*

*A lot of times, I have patients who just muddle through because they're afraid that they'll lose their job, or their boss won't understand.*

Suspicion of invisible conditions, while not uncommon, is particularly challenging with an injury like mTBI that is so variable and unpredictable, even for medical professionals. The recent and multifaceted surge in advocacy and awareness raising in sports, media, and the military, however, promises to shift this trend, if slowly.

## 4. Conclusions

Rehabilitation with mTBI is complex and challenging work that requires professionals to address a range of psychosocial issues faced by their patients. Individuals with mTBI often had prior experience with misdiagnosis and misinformation and turned to their rehabilitation specialists for far more than standard rehabilitation services. Many sought clarification about their complex symptomology with the hope of also getting insight on a prognosis. Although unaware, at times, of their own limitations resulting from the trauma that was sustained, many patients were keenly aware of how the invisible nature of mTBI caused others to question the legitimacy of their injury.

This study suggests a need for further research in this area. It seems evident that mTBI rehabilitations is unexpectedly challenging for both workers and patients given the expectations associated with a “mild” injury. More information is needed about the emotional energy required to manage a caseload of mTBI patients, with specific attention to the type of training that rehabilitation professionals need to do this work both effectively and ethically. Lastly, although the trauma of mTBI refers to the external nature of the source of injury, this study suggests there may also be a traumatic element in the rehabilitation work as frontline professionals vicariously experience their patients' challenging and complex recoveries.
